# Treatment of localised resectable neuroblastoma. Results of the LNESG1 study by the SIOP Europe Neuroblastoma Group

**DOI:** 10.1038/sj.bjc.6604640

**Published:** 2008-09-02

**Authors:** B De Bernardi, V Mosseri, H Rubie, V Castel, A Foot, R Ladenstein, G Laureys, M Beck-Popovic, A F de Lacerda, A D J Pearson, J De Kraker, P F Ambros, Y de Rycke, M Conte, P Bruzzi, J Michon

**Affiliations:** 1 Department of Paediatric Haematology and Oncology, Giannina Gaslini Children's Hospital, Genova, Italy; 2 Statistical Unit, Institut Curie, Paris, France; 3 Department of Paediatric Haematology and Oncology, Hôpital des Enfants, Toulouse, France; 4 Paediatric Oncology Unit, Hospital Infantil Universitario La Fe, Valencia, Spain; 5 Department of Paediatric Oncology, Royal Hospital for Children, Bristol, UK; 6 Department of Paediatric Oncology, St Anna Kinderspital, Vienna, Austria; 7 Department of Paediatric Haematology and Oncology, University Hospital, Ghent, Belgium; 8 Department of Paediatric Haematology and Oncology, Centre Hospitalier Universitaire Vaudois, Lausanne, Switzerland; 9 Department of Pediatrics, Intituto Português de Oncologia Francisco Gentil, Lisboa, Portugal; 10 Department of Paediatric Haematology and Oncology, Royal Marsden Hospital, Sutton, UK; 11 Department of Paediatric Oncology, Emma Children's Hospital/Academic Medical Centre, Amsterdam, The Netherlands; 12 Childrens' Cancer Research Institute, St Anna Kinderspital, Vienna, Austria; 13 Clinical Trials Unit, National Cancer Institute, Genova, Italy; 14 Department of Paediatrics, Institut Curie, Paris, France

**Keywords:** neuroblastoma, localised, *MYCN* gene, prognostic factors

## Abstract

Main objective of this study was to confirm that surgery alone is an effective and safe treatment for localised resectable neuroblastoma except stage 2 with amplified *MYCN* gene (MYCNA). Of 427 eligible stages 1–2 patients, 411 had normal *MYCN* and 16 had MYCNA. Of the 288 stage 1 patients with normal *MYCN*, 1 died of complications and 16 relapsed, 2 of whom died; 5-year relapse-free survival (RFS) and overall survival (OS) rates were 94.3% (95% confidence interval (CI): 91.6–97) and 98.9% (95% CI: 97.7–100), respectively. Of the 123 stage 2 patients with normal *MYCN*, 1 died of sepsis and 22 relapsed, 8 of whom died (RFS 82.8%, 95% CI: 76.2–89.5; OS 93.2%, 95% CI: 88.7–97.8). In stage 2, OS and RFS were worse for patients with elevated LDH and unfavourable histopathology. Of 16 children with MYCNA, 7 were stage 1 (5 relapses and 4 deaths) and 9 were stage 2 (3 relapses and 2 deaths) patients. In conclusion, surgery alone yielded excellent OS for both stage 1 and 2 neuroblastoma without MYCNA, although stage 2 patients with unfavourable histopathology and elevated LDH suffered a high number of relapses. Both stage 1 and 2 patients with MYCNA were at greater risk of relapse.

Neuroblastoma, the most common extracranial solid tumour in childhood, originates from the primitive cells of the sympathetic nervous system (Caron and Pearson, 2005). Approximately half of the children diagnosed with neuroblastoma have metastatic disease on diagnosis and, except for infants (De Bernardi *et al*, 1992), have a low probability of cure (De Bernardi *et al*, 2003). The remaining children present with localised disease and have an overall better prognosis, mainly depending on the degree of tumour resection (Cecchetto *et al*, 1983; von Schweinitz *et al*, 2000). Indeed, completely resected tumours (INSS stage 1) (Brodeur *et al*, 1993) rarely relapse and do not usually undergo postoperative chemotherapy (Kushner *et al*, 1996; Alvarado *et al*, 2000). By contrast, localised but unresectable neuroblastomas (INSS stage 3) require primary chemotherapy before surgical excision is attempted, and in any case have a greater likelihood of relapse and a lower cure rate (Garaventa *et al*, 2002).

An intermediate condition involves primary tumour resection that has left behind a small residue (INSS stage 2). Some authors have suggested leaving these tumour residues untreated (De Bernardi *et al*, 1995; Perez *et al*, 2000); however, some of these tumours relapse (occasionally in a disseminated form) and then require intensive treatment, which is sometimes unsuccessful. At present, only abnormal *MYCN* gene status is believed to correlate with worse outcome in these patients, and postoperative treatment is therefore deemed necessary only in such cases (Rubie *et al*, 1997). A number of other factors, including regional lymph node involvement (Ninane *et al*, 1982; Rosen *et al*, 1984), tumour rupture (De Bernardi *et al*, 1995), unfavourable histology (Perez *et al*, 2000; Shimada *et al*, 2001), DNA index (Bagatell *et al*, 2005), and some biochemical markers (Hann *et al*, 1985; Zeltzer *et al*, 1986; Shuster *et al*, 1992), have been associated with greater risk of relapse and death.

In the early 1990s, the SIOP Europe Neuroblastoma Group (SIOPEN) activated the first Localised Neuroblastoma European Study (LNESG1). The principal objectives of this study were to confirm that surgery alone suffices in all stage 1 children and to assess whether the same is true for stage 2 children with normal *MYCN* gene status. A secondary objective of the LNESG1 study was to establish a European registry for all localised neuroblastomas.

In this report, we describe the outcome of both stage 1 and 2 patients stratified according to *MYCN* gene status.

## Patients and methods

### Patient registration and eligibility

The registration of children with suspected, localised neuroblastoma potentially eligible for the LNESG1 Study began in January 1995 and ended in October 1999. In an attempt to ensure the enrolment of children who had suffered major surgery-related complications, the investigators were requested to register all suspected cases with non-metastatic neuroblastoma before histological confirmation. Data were collected at the Study Coordinating Centre, located at the Institut Curie, Paris, France. Nine European countries (Austria, Belgium, France, Italy, The Netherlands, Portugal, Spain, Switzerland, and United Kingdom) participated in the study.

To be eligible in the study, patients had to fulfil all of the following criteria: age on diagnosis below 20 years, histological diagnosis of neuroblastoma (excluding ganglioneuroma), evaluation of *MYCN* gene status, primary tumour resectability, the absence of metastasis, and no previous therapy. Informed consent was provided by a parent or guardian for each patient enrolled in the study. Study approval was obtained from the local institutional review boards.

### Diagnostic procedures and metastatic workup

The diagnostic imaging of the tumour included ultrasound scan plus computed tomography and/or magnetic resonance imaging (Siegel *et al*, 2002). These investigations were carried out in an effort to detect features (defined as surgical risk factors) that are believed to reduce the probability of complete tumour resection and that lead to increased risk of surgery-related complications. Immediate surgery was recommended for patients without surgical risk factors, whereas the remaining patients were to receive chemotherapy in preparation for surgery. Details regarding surgical risk factors and surgical guidelines have been reported elsewhere (Cecchetto *et al*, 2005).

Bone marrow infiltration was ruled out on the basis of four negative specimens (two bone marrow aspirates and two trephine core biopsies for children aged 1 year or more; 2–4 aspirates for infants). Skeletal involvement was evaluated by MIBG (iodine-123-metaiodobenzylguanidine scintigraphy) or by technetium-99m-MDP scintigraphy in the absence of MIBG uptake or if MIBG scintigraphy had not been performed prior to surgery. Involvement of other organs, mainly the liver, lung, skin, and distant lymph nodes, was studied by ultrasound scan and/or computed tomography.

Biochemical studies included the assay of catecholamine metabolites, that is vanillylmandelic acid and homovanillic acid in the urine (Strenger *et al*, 2007), and lactate dehydrogenase (LDH) assay in the serum. Vanillylmandelic acid, homovanillic acid, and LDH levels were considered to be abnormal if they exceeded twice the upper normal value.

### Histopathology

Histological diagnosis was performed by the local pathologists according to the Shimada system and the related classification (Shimada *et al*, 1999, 2001). Non-*MYCN* amplified stage 2 tumours were reviewed by an European Pathology Panel (Navarro *et al*, 2006).

### Biological studies

To guarantee the reproducibility of the results, 11 laboratories from 9 European countries set up an European Neuroblastoma Quality Assessment (ENQUA) Group (Ambros *et al*, 2003). This led to the establishment of common rules with regard to the handling of tumour materials, methods, and DNA probes to be used, and the interpretation of DNA blots and fluorescent *in situ* hybridisation (FISH) analyses. The evaluation of *MYCN* gene status in tumour cells was a prerequisite to enrolling patients in the study. *MYCN* gene amplification (MYCNA) was defined as a copy number of 10 or more, or a greater than fourfold increase in the *MYCN* signal number in comparison with the reference probe located on chromosome 2. Evaluation of DNA content was strongly recommended.

### Adjuvant therapy

Stage 1 patients, regardless of *MYCN* gene status, and stage 2 patients without MYCNA received no adjuvant therapy (except for those with symptomatic spinal cord compression) (De Bernardi *et al*, 2005) and were carefully followed up. Stage 2 patients with MYCNA were treated according to institutional guidelines.

### Follow-up

Computed tomography or magnetic resonance imaging was performed within 1 month of surgery to assess the degree of the residual tumour, if any. Patients were subsequently evaluated by clinical examination and ultrasound scan at least once every 3 months in the first year, and every 3–6 months over the following 5 years.

### Tumour relapse and progression

In the event of relapse or progression, complete disease evaluation was carried out, including histological (or cytohistological) examination, assay of *MYCN* status, and metastatic workup. Local relapse was preferentially treated by surgery and standard-dose chemotherapy according to national protocols. Metastatic relapse was treated according to the current national stage 4 protocols.

### Statistical analyses

The prospective European registration of localised neuroblastoma was activated in January 1995. Stage 1, 2 and 3 subjects were to be registered. According to LNESG1 protocol, 140 stage 2 patients without MYCNA were to be enrolled in this trial that involved surgery as the only treatment in an effort to assess whether the conservative approach alone was enough to keep the 3-year survival rate above 90%. Enrollment was expected to last 3.5 years. Four and a half years after the beginning of the study (which lasted from January 1995 to October 1999), a total of 123 stage 2 patients had been recruited, representing 16.7% of all localised neuroblastomas. At that time, the trial, as well as the prospective registration, was closed. The frequency of clinical, biological, and pathological features in various subgroups was compared by the *χ*^2^ test or by two-tailed Fisher's exact test when necessary.

Overall survival and relapse-free survival (RFS) curves were estimated according to the Kaplan–Meier method and compared by log-rank test, and were calculated from the day of tumour resection. Overall survival took all deaths into account. In RFS, local or distant recurrences were considered as events. Patients who did not relapse were censored at the time of death or last follow-up.

## Results

Between January 1995 and October 1999, a total of 905 children and adolescents with suspected localised neuroblastoma were registered in the LNESG1 Study by 107 European institutions. Of these, 215 children were excluded for various reasons: inadequately assessed *MYCN* gene (95 cases), staging errors (47), wrong diagnosis (28), previous treatment (13, including 5 stage 2 patients presenting with symptomatic spinal cord compression), undefined histology (9), parental refusal (5), and other reasons (18) (Table 1). Of the 690 children with non-metastatic neuroblastoma, 192 had stage 3 disease and 71 had a diagnosis of ganglioneuroma, leaving 427 eligible children with localised resectable neuroblastoma (Table 1). Out of these 427 children, 411 (288 stage 1 and 123 stage 2) had normal *MYCN* gene status, whereas 16 (7 stage 1 and 9 stage 2) had MYCNA (Table 1). The following results refer to the stage 1 and 2 patients stratified as per *MYCN* status.

### Stage 1 and 2 patients without MYCNA

#### Patients’ characteristics at diagnosis

The main presenting characteristics of the 288 stage 1 and the 123 stage 2 children are listed in Table 2. There was a slight male predominance (M/F ratio, 1.4) among stage 1 patients, whereas the number of stage 2 males and females was almost the same. The median age of both stage 1 and stage 2 patients was 11 months, with similar percentages of patients in the two age groups considered (0–17, ⩾18 months). Stage 1 patients had more abdominal tumours than stage 2 patients (68.4 *vs* 49.6%; *P*<0.0025), and they more often had favourable histological features, as evaluated by the local pathologists (88.4 *vs* 79.8%; *P*<0.04), normal vanillylmandelic acid (67.4 *vs* 44.4%; *P*=0.0008) and normal homovanillic acid urinary excretion (67.5 *vs* 50.7%; *P*=0.015). The percentages of normal *vs* abnormal LDH serum levels (93.1 *vs* 92.2%) and aneuploid *vs* di-tetraploid DNA index (64.3 *vs* 61.8%) were similar.

#### Surgery-related complications

Among the 411 stage 1 and 2 patients, 1 (a stage 1 infant) died of surgery-related complications 2 days after the operation, owing to massive bleeding and multiple organ failure. Thirty patients (7.3%) – 17 out of 288 stage 1 (5.9%) and 13 out of 123 stage 2 subjects (10.6%) – developed non-fatal complications: Horner's syndrome (6 cases), pleural effusion or ascitis (5), renal ischaemia or damage (4), intestinal perforation (3), serious bleeding (2), vascular damage (2), severe infections (2), tracheostomy (1), neurological damage (1), and others (4).

#### Clinical course and survival

**Figure 1:**
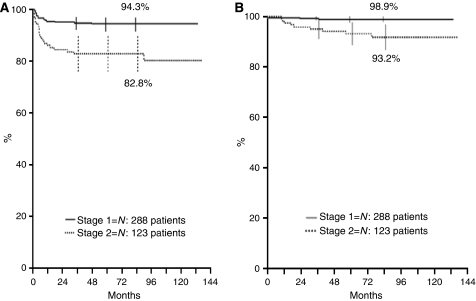
Five-year RFS (**A**) and OS (**B**) according to INSS stage.

*Stage 1.* Patient follow-up ranged from 3 to 134 months (median, 72). Of the 288 patients, 1 died shortly after surgery (see above), and 16 relapsed 1–48 months after diagnosis (median, 5) (Table 3); the estimated 5-year RFS rate was therefore 94.3% (95% confidence interval (CI): 91.6–97.0) (Figure 1A). Relapses occurred at the site of the primary tumour in five cases, at distant sites in seven, and both locally and at distant sites in four cases. Sites of metastases included various combinations of the following: bone (six cases), bone marrow (five), and liver and skin (three cases each). Among the 10 patients who had been diagnosed between 0 and 11 months of age, relapses occurred locally, or in the liver, skin, or bone marrow, and were interpreted as a possible evolution towards stage 4. Five of these patients were simply observed, whereas five were treated with a few cycles of chemotherapy; all survived (Table 3). The remaining six patients who relapsed were older than 1 year on diagnosis; two of these died (one of progressive disease and one of chemotherapy-related toxicity) and four survived (Table 3). The estimated 5-year OS was 98.9% (95% CI: 97.7–100) (Figure 1B). The 5-year OS of the 16 patients who relapsed was 87.5% (95% CI: 71.3–100).

*Stage 2*. Patient follow-up ranged from 10 to 137 months (median, 80). Of the 123 patients, 1 died of sepsis due to surgery-related spleen atrophy 18 months after tumour resection, and 22 relapsed 1–90 months after diagnosis (median, 6) (Table 4); the estimated 5-year RFS rate was therefore 82.8% (95% CI: 76.2–89.5) (Figure 1A). Relapses occurred locally in 13 cases, at distant sites in 1, and both locally and at distant sites in 8. Sites of metastases included various combinations of the following: bone (five cases), bone marrow (five), liver and lung (one case each) (Table 4). The median interval between diagnosis and relapse was 6 months, both in cases of local relapse and in those of relapse at distant sites. Of the 12 patients below 1 year of age on diagnosis who relapsed, 2 died, whereas 6 deaths occurred among the 10 patients aged 1 year or older. The eight deaths occurred 5–55 months after relapse, with no difference between patients who relapsed locally and those who relapsed at distant sites (30 months for both). The estimated 5-year OS was 93.2% (95% CI: 88.7–97.8) (Figure 1B). The 5-year OS of the 22 patients who relapsed was 56.1% (95% CI: 32.5–79.7).

#### Prognostic factors

Analyses aimed at assessing the impact of potential prognostic factors on OS and RFS were limited by the small number of events. Among stage 1 patients, no analysis of OS was possible (three events). Regarding RFS, the only prognostic factor we identified was gender, with males relapsing more often than females (5-year RFS 92.0 *vs* 97.5%; *P*<0.05) (Table 5). Normal LDH serum levels were associated with greater RFS (95.6 *vs* 83.3%), but the difference was not statistically significant (*P*=0.061). Among stage 2 patients, no noticeable differences in either OS or RFS were observed with regard to gender, age, lymph node infiltration, vanillylmandelic acid and homovanillic acid urinary excretion, or DNA index (Table 5). Patients with unfavourable histopathologic features had significantly worse OS and RFS than those with favourable features (96.4 *vs* 75.9%, *P*<0.0004; and 85.5 *vs* 61.2%, *P*<0.0035, respectively) (Table 5). In addition, normal LDH serum levels were significantly associated with better OS and RFS (95.7 *vs* 50.0%; *P*<0.0001; 85.2 *vs* 62.5%; *P=*0.050, respectively) (Table 5).

### Stage 1 and 2 with MYCNA

Of the 427 children with localised resectable neuroblastoma who were evaluated for *MYCN* gene status, 16 (3.7%) had abnormal copy numbers of the gene, including 7 stage 1 and 9 stage 2 patients. Five of the seven stage 1 patients suffered relapses: four local and one both local and in the bone and bone marrow. Four of these five patients eventually died. Of the nine stage 2 patients who received upfront chemotherapy according to the guidelines of the individual participating institutions, three relapsed, all locally and at distant sites; two eventually died.

## Discussion

This study focused on localised, resectable tumours and aimed at assessing whether surgery alone is an adequate and safe treatment for all stage 1 patients and for stage 2 patients without MYCNA (Rubie *et al*, 1997). Overall, 411 out of 905 enrolled patients were classified as either INSS stage 1 or stage 2 without MYCNA. The 288 stage 1 patients in this series did very well and had an OS of 98.9%; this is in line with previous literature reports (De Bernardi *et al*, 1995; Evans *et al*, 1996; Kushner *et al*, 1996; Alvarado *et al*, 2000; Perez *et al*, 2000), and confirms that neuroblastomas that are resected macroscopically and have normal *MYCN* status do not need adjuvant therapy and are commonly salvageable in the event of relapse. Indeed, only one of three deaths was due to disease progression, whereas the other two were related to treatment. Physicians should therefore avoid letting risks that are secondary to therapy exceed the risks related to this scarcely aggressive neoplasm. It is noteworthy that, of the 16 events recorded in these 288 stage 1 patients, all the 10 that occurred in infants did well, although 5 of them underwent observation only after the event had occurred. This fact was interpreted as the possible evolution towards stage 4S disease on the basis of the very young age (less than 6 months in 7 out of 10) and the sites of progressions (mainly the skin, bone marrow, and primary tumour). This is possibly the first observation of this type and may reflect the increased ability to detect stage 4S disease at an early phase, that is before the multifocal nature of the tumour has become evident. Unlike the other stage 1 patients, the seven with MYCNA who also underwent a wait-and-see strategy tended to have an unfavourable course; indeed, five of them relapsed and four eventually died, suggesting that adjuvant chemotherapy should be seriously considered for this rare population. A pooled analysis of existing evidence is warranted to clarify this issue.

Of 123 stage 2 patients without MYCNA who were enrolled in this study and who underwent observation alone after surgery, 1 died of surgery-related complications and 22 relapsed, 8 of whom died. Relapses occurred locally in almost all cases, with associated metastatic spread in about half of them, thus resulting in an RFS rate of 82.8%. The risk of relapse was significantly greater in association with unfavourable histological features, confirming the findings of other authors (Perez *et al*, 2000; Shimada *et al*, 2001), and elevated LDH serum levels, although in the latter case the small number of events precludes firm conclusions about the prognostic significance of this parameter. However, at variance with previous reports, the risk of relapse did not correlate significantly with the age at diagnosis (De Bernardi *et al*, 1995), regional lymph node infiltration (Ninane *et al*, 1982), and DNA index (Bagatell *et al*, 2005). These conflicting results may be accounted for by the small number of cases in the published series. Eight of the 22 stage 2 patients who relapsed died of disease, and the risk of progression and death was independent of the extent of relapse. Although the 5-year OS of 93.2% is in line with previous literature data (Matthay *et al*, 1989; De Bernardi *et al*, 1995; Perez *et al*, 2000), it indicates that, despite having a normal *MYCN* gene status, a non-negligible proportion of these patients are candidates for a worrisome clinical course and may die of disease progression. Clearly, further biological studies are needed to identify which patients might benefit from adjuvant treatment.

Interestingly, one-third of the nine stage 2 patients who had MYCNA and therefore received adjuvant therapy on an institutional basis relapsed and only two eventually died (22%), as against the four deaths (57%) observed among the seven stage 1 patients with MYCNA who did not receive adjuvant chemotherapy. Although the small number of patients available for these analyses precludes any firm conclusions, our findings underline the need for studies aimed at evaluating the hypothesis that immediate postoperative chemotherapy may effectively act against the minimal post-surgical tumour residue in this particular setting.

## Figures and Tables

**Table 1:** Patients in the LNESG1 study

		**No of patients**
Enrolled		905
*Excluded*		215
Inadequate *MYCN* gene assay	95	
Staging errors	47	
Wrong diagnosis	28	
Undefined histology	9	
Previous treatment	13	
Parental refusal	5	
Other reasons	18	
		
*Non-metastatic neuroblastoma*		690
Ineligible for the study		263
Stage 3	192	
Ganglioneuroma	71	
Eligible for the study		427
Study 1		295
Without MYCNA	288	
With MYCNA	7	
Study 2		132
Without MYCNA	123	
With MYCNA	9	

LNESG1=Localised Neuroblastoma European Study.

**Table 2:** Stage 1 and 2 patients without MYCNA. Characteristics at diagnosis

	**Stage**						
	**1 (*n*=288)**			**2 (*n*=123)**			
**Characteristics**	**No. of patients**	***%***	**95% CI**	**No. of patients**	***%***	**95% CI**	***P*-value**
*Gender*							
Male	167	58	52.3–63.7	61	49.6	40.8–58.4	0.11
Female	121	42	36.3–47.7	62	50.4	41.6–59.2	
M/F ratio		1.4			1		
							
*Age (months)*							0.63
Median		11		11			
0–17	185	64.2	58.7–69.8	76	61.8	53.2–70.4	
>18	103	35.8	30.2–41.3	47	38.2	29.6–46.8	
							
*Site of primary tumour*							<0.0025
Neck	14	4.9	2.4–7.3	7	5.7	2.3–11.4	
Thorax	65	22.5	17.7–27.4	43	35	26.5–43.4	
Abdomen	197	68.4	63–73.8	61	49.6	40.8–58.4	
Pelvis	12	4.2	1.9–6.5	12	9.7	4.5–15	
							
*Urine VMA (247 tested)*							0.0008
Normal	118	67.4	60.5–74.4	32	44.4	33–55.9	
Elevated	57	32.6	25.6–39.5	40	55.6	44.1–67	
							
*Urine HVA (231tested)*							0.015
Normal	108	67.5	60.2–74.8	36	50.7	39.1–62.3	
Elevated	52	32.5	25.2–39.8	35	49.3	37.7–60.9	
							
*Serum LDH (276 tested)*							0.79
Normal	161	93.1	89.3–96.8	95	92.2	85.3–96.6	
Elevated	12	6.9	3.1–10.7	8	7.8	3.4–14.7	
							
*Histopathologic category (320 tested)*							<0.04
Favourable	191	88.4	84.2–92.7	83	79.8	72.1–87.5	
Unfavourable	25	11.6	7.3–15.8	21	20.2	12.5–27.9	
							
*DNA index (216 tested)*							0.72
Aneuploid	90	64.3	56.3–72.2	47	61.8	50.9–72.8	
Di-tetraploid	50	35.7	27.8–43.6	29	38.2	27.2–49.1	

HVA=homovanillic acid; LDH=lactate dehydrogenase; VMA=vanillylmandelic acid.

**Table 3:** Relapses/progressions in 16 stage 1 patients without MYCNA

**Case no.**	**Age (months)**	**Primary site**	**Histopathology**	**LDH**	**Ploidy**	**Site/time to relapse/progression**	**Therapy**	**Outcome**
1	0	A	—	E	—	Skin, 4 months	None	CR, 81 months
2	1	A	F	N	—	B, Skin, L, 3 months	None	CR, 74 months
3	1	Neck	F	—	An	Local, 3 months	CT	CR, 122 months
4	1	A	F	—	An	Local, 10 months	CT	CR, 87 months
5	2	A	F	N	—	L, 2 months	CT	CR, 62 months
6	2	A	F	N	An	B, Skin, L, 5 months	None	CR, 67 months
7	5	A	F	N	D	Local, B, 11 months	None	CR, 58 months
8	7	A	F	N	An	BM, 1 month	CT	CR, 117 months
9	10	T	F	N	An	Local, 3 months	None	CR, 112 months
10	11	A	F	N	D	Local, 48 months	CT, RT	CR, 92 months
11	26	T	F	—	An	Local, 23 months	CT, S	CR, 37 months
12	26	A	U	N	An	B, BM, 3 months	CT, S	CR, 127 months
13	52	A	—	E	D	BM, 11 months	None	CR, 70 months
14	59	A	—	N	—	Local, B, 10 months	CT	CR, 89 months
15	71	T	—	—	D	Local, BM, 5 months	CT	DOD, 24 months
16	169	A	—	—	—	Local, B, BM, 35 months	CT, RT	TD, 38 months

A=abdomen; An=aneuploid; B=bone; BM=bone marrow; CR=complete remission; CT=chemotherapy; D=diploid; DOD=dead of disease; E=elevated; F=favourable; L=liver; N=normal; RT=radiotherapy; S=surgery; T=thorax; TD=toxic death; U=unfavourable.

**Table 4:** Relapses/progressions in 22 stage 2 patients without MYCNA

**Case no.**	**Age (months)**	**Primary site**	**INSS stage**	**Histopathology**	**LDH**	**Ploidy**	**Site/time to relapse/progression**	**Therapy**	**Outcome**
1	0	P	2A	U	N	An	Local, 6 months	None	CR, 134 months
2	0	P	2B	F	—	—	Local, 2 months	CT	LFU, 20 months
3	1	A	2B	F	N	An	Local, 5 months	CT	DOD, 10 months
4	2	A	2A	F	N	An	Local, 1 month	CT	DOD, 10 months
5	2	T	2A	F	N	Te	Local, 4 months	CT	CR, 105 months
6	2	A	2B	F	N	D	Local, BM, 7 months	CT	CR, 88 months
7	3	A	2B	U	N	An	Local, BM, 8 months	CT, S	CR, 112 months
8	3	P	2A	F	—	—	Local, Skin 6 months	CT, S	CR, 125 months
9	6	A	2B	F	N	An	Local, 14 months	CT	CR, 88 months
10	9	P	2B	F	N	An	Local, 13 months	CT	CR, 38 months
11	10	A	2B	F	N	An	B, BM, 6 months	CT, RT	LFU, 27 months
12	10	A	2A	F	N	Te	Local, 9 months	RT	Unknown
13	13	A	2A	—	—	—	Local, 15 months	CT	CR, 55 months
14	14	Neck	2B	F	—	An	Local, 1 month	CT, S	CR, 89 months
15	27	A	2A	U	E	Te	Local, B, Lung, 2 months	CT, S	DOD, 27months
16	42	T	2A	U	N	Te	Local, BM, B, 4 months	CT, S	DOD, 46 months
17	43	A	2B	U	E	Te	Local, 1 month	CT, S	DOD, 57 months
18	47	T	2B	F	N	—	Local, BM, 3 months	CT, S	DOD, 19 months
19	55	A	2B	U	N	—	Local, 36 months	CT, S	DOD, 77 months
20	65	A	2B	U	E	D	Local, B, 5 months	S, CT	DOD, 32 months
21	68	T	2A	U	N	—	Local, 26 months	None	CR
22	100	P	2B	U	N	D	Local, B, Liver, 92 months	None	LFU, 93 months

A=abdomen; An=aneuploid; B=bone; BM=bone marrow; CR=complete remission; CT=chemotherapy; D=diploid; DOD=dead of disease; E=elevated; F=favourable; LFU=lost to follow-up; N=normal; P=pelvis; RT=radiotherapy; S=surgery; T=thorax; Te=Tetraploid; U=unfavourable.

**Table 5:** Five-year OS and RFS for stage 1 and stage 2 patients without MYCNA

	**Stage 1**							**Stage 2**						
**Characteristics**	**Cases**	**No. of deaths**	**OS**	***P*-value**	**No. of events**	**RFS**	***P*-value**	**Cases**	**No. of deaths**	**OS**	***P*-value**	**No. of events**	**RFS**	***P*-value**
	288	3	98.9		16	94.3		123	9	93.2		22	82.8	
*Gender*							<0.05				0.79			0.16
Male	167				13	92		61	4	93.2		8	88.5	
Female	121				3	97.5		62	5	93.4		14	77.4	
														
*Age (years)*							0.91				0.07			0.90
0–17	185				10	94.5		76	3	96.0		14	81.5	
⩾18	103				6	94.1		47	6	88.7		8	85.0	
														
*Primary site*							0.79				0.45			0.076
Neck	14				1	92.9		7	1	100		2	71.4	
Thorax	65				3	95.4		43	2	95.3		4	90.7	
Abdomen	197				12	93.7		61	6	89.6		11	81.8	
Pelvis	12				0	100		12	0	100		5	66.7	
														
*LN infiltration*											0.50			0.79
Absent	0							53	3	94.3		9	83.0	
Present	0							70	6	92.4		13	82.7	
														
*Serum LDH*							0.061				<0.0001			0.050
Normal	161				7	95.6		95	5	95.7		15	85.2	
Increased	12				2	83.3		8	4	50		3	62.5	
Missing	115							20						
														
*Urine VMA*							0.44				0.20			0.87
Normal	118				5	95.8		32	3	90.4		6	84.4	
Increased	57				4	92.8		40	1	97.5		7	82.5	
Missing	113							51						
														
*Urine HVA*							0.28				0.31			0.39
Normal	108				4	96.3		36	3	91.4		8	80.6	
Increased	52				4	92.1		35	1	97.1		5	85.7	
Missing	128							52						
														
*Histopathologic category*							0.78				<0.0004			<0.0035
Favourable	191				10	94.7		83	3	96.4		12	85.5	
Unfavourable	25				1	96		21	6	75.9		9	61.2	
Missing	72							19						
														
*DNA index*							0.40				0.31			0.40
Aneuploid	90				7	92.2		47	3	93.6		8	83	
Di-tetraploid	50				4	91.5		29	4	85.9		7	79.3	
Missing	148							47						

HVA=homovanillic acid; LDH=lactate dehydrogenase; LN=lymph node; OS=overall survival; RFS=relapse-free survival; VMA=vanillylmandelic acid.
